# Blood Supply of Cranial Nerves Passing Through the Cavernous Sinus: An Anatomical Study and Its Implications for Microsurgical and Endoscopic Cavernous Sinus Surgery

**DOI:** 10.3389/fonc.2021.702574

**Published:** 2021-10-08

**Authors:** Edinson Najera, Bilal Ibrahim, Baha’eddin A. Muhsen, Assad Ali, Clariza Sanchez, Michal Obrzut, Hamid Borghei-Razavi, Badih Adada

**Affiliations:** Cleveland Clinic Florida, Weston, United States

**Keywords:** intracavernous cranial nerves, inferolateral trunk, meningohypophyseal trunk, oculomotor nerve, trochlear nerve, abducens nerve, trigeminal nerve

## Abstract

**Background:**

Despite improvements in surgical techniques, cranial nerve (CN) deficits remain the most frequent cause of disability following cavernous sinus (CS) surgery. The most common tumor affecting the CS is meningioma. They originate from lateral wall and have their blood supply from meningohypophyseal trunk (MHT) and inferolateral trunk (ILT). Pituitary adenomas commonly invade the CS through its medial wall and receive blood supply form medial branches of the internal carotid artery (ICA) (superior and inferior hypophyseal arteries). Some tumors may grow within the CS (e.g. trigeminal schwannomas, hemangiomas). These tumors are fed by all the intracavernous ICA branches. Tumors involving the CS may also displace the neurovascular structures, therefore, a better understanding of intracavernous neurovascular anatomy may reduce the postoperative morbidity associated with approaching CS tumors. In this anatomical study, the anatomic variations and their clinical implications of the intracavernous CNs’ blood supply were evaluated through transcranial and endonasal routes.

**Methods:**

Twenty sides of ten adult cadaveric formalin-fixed, latex-injected specimens were dissected in stepwise fashion under microscopic and endoscopic magnification. The origin and course of the intracavernous ICA branches supplying the intracavernous CNs are studied.

**Results:**

The proximal segment of the oculomotor nerve receives blood supply from the ILT in 85%, and the tentorial artery of the MHT in 15% of specimens. The distal segment is exclusively supplied by the ILT. The proximal trochlear nerve receives blood supply from the ILT (75%) and the tentorial artery (25%); the distal segment is exclusively supplied by the superior orbital branch. The proximal third of the abducens nerve receives its vascularity exclusively from the dorsal meningeal artery, and its middle and distal thirds from the ILT. The ophthalmic and proximal maxillary segments of the trigeminal nerve also receive blood supply from the ILT. The distal maxillary segment is supplied by the artery of the foramen rotundum. All ILT branches terminate on the inferomedial aspects of the intra-cavernous CNs. Extensive anastomoses are found between ILT branches and the branches arising from external carotid artery.

**Conclusion:**

Understanding the anatomy of the intracavernous ICA’s branches is important to improving surgical outcomes with tumors involving the CS.

## Introduction

Tumors with cavernous sinus (CS) invasion present a neurosurgical challenge. The most common tumor seen in cavernous sinus is meningioma (1). However, many tumors can arise in or invade the CS, these include trigeminal schwannomas, pituitary adenomas, chordomas and chondrosarcomas. Advances in cranial base surgery over the last two decades including better anatomical understanding, improvement in microsurgical technics and incorporation of the endonasal endoscopic approach (EEA) into the skull base armamentarium, now allow for 360° access to the CS. Despite the improvements in microscopic and endoscopic surgical techniques, cranial nerve (CN) deficits remain the most frequent cause of disability following CS surgery (1–3). Nerve dysfunction can occur due to direct trauma caused by the manipulation of the nerve during surgery or from ischemic injury caused by damage to the nerve’s blood supply. Permanent CN deficits may occur even if the nerves are anatomically preserved (1, 2, 4). Thorough knowledge in the anatomy of blood vessels supplying CNs inside the CS is necessary for approaching tumors with expansion in to the CS. This understanding is particularly important to minimize inadvertent occlusion of supplying blood vessels from excessive coagulation or manipulation during tumor resection. The branches of the intracavernous internal carotid artery (ICA) have been described by several authors, mostly from the microsurgical transcranial perspective (1, 5–11). There are only a few descriptions providing a detailed anatomy of the blood supply to the intracavernous CNs from both a transcranial and an EEA perspective (12–14).

In this anatomical study, we evaluate the anatomic variations and clinical implications of the intracavernous CNs’ blood supply when approaching the CS region from transcranial and endonasal routes. A detailed understanding of the intracavernous CNs’ blood supply is essential for improving outcomes when operating on lesions involving the CS.

## Methods

Ten adult head specimens (20 sides) were lightly fixed in a formalin solution and prepared for dissection after injecting them with intravascular colored silicone. This research was approved by the Research Ethics Board. A microsurgical transcranial approach and an EEA to the sellar and parasellar regions were performed simultaneously on all heads. A pterional craniotomy was performed, followed by extradural drilling of the anterior clinoid process. Then, a 5-cm temporal lobectomy was performed, and the lateral, posterior wall of the CS was exposed. Under microscopic magnification, the dura covering the lateral wall of the CS was removed, exposing the CNs in the lateral wall of the sinus. From the EEA perspective, wide bilateral sphenoidotomies were performed to expose the posterior wall of the sphenoid sinus. The bone overlying the sellar and parasellar regions was removed to allow access to the medial and anterior walls of the CS. The origin, course, and different anatomic patterns of the intracavernous ICA branches vascularizing the intracavernous CNs were documented from both the transcranial and the EEA perspectives. CNs are then followed proximal and distal to CS segments and vascularizing branches to these segments were studied and documented. A tumor with CS invasion was selected to illustrate the surgical application of these findings.

## Results

### The Intracavernous ICA and Its Branches

The arterial branches arising from the intracavernous segment of the ICA vary in origin and number. The intracavernous ICA from proximal to distal can be divided into four segments: 1) the short vertical segment (a continuation of the paraclival ICA); 2) the posterior genu; 3) the horizontal segment; and 4) the anterior genu, which continues with the paraclinoidal ICA as it emerges from the CS. The most common branches of the intracavernous ICA observed in this study are the meningohypophyseal trunk (MHT) (present in 100% of specimens), the inferolateral trunk (ILT) (85%) and McConnell’s capsular artery (20%). Less frequent branches of the intracavernous ICA are the superolateral trunk (10%) and a persistent trigeminal artery (5%).

**Figure 1:**
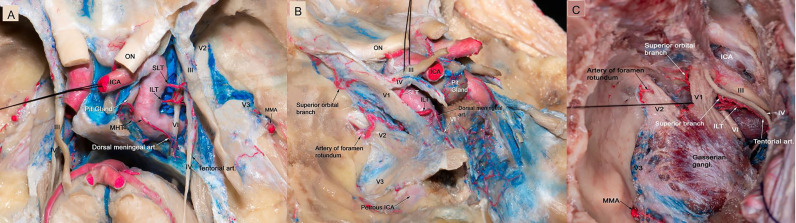
**(A)** Microscopic view of the roof and lateral wall of the CS in a colored silicone-injected human cadaveric specimen showing the branches of the intracavernous ICA supplying the intracavernous CN. The dural covering of the right cavernous sinus has been removed to expose the cavernous sinus contents. The MHT originating from the proximal cavernous segment of the ICA, in this specimen a double trunk is present, the main trunk is giving off the dorsal meningeal artery, which passed posteriorly through the CS to supply the clival dura and abducens nerve within Dorello’s canal. The ILT arose from the lateral side of the midportion of the horizontal segment of the intracavernous ICA, and is directed inferiorly. It provides blood supply to CN III, CN IV, middle and distal segments of CN VI. The SLT arises from the supero-lateral aspect of the horizontal segment of the intracavernous ICA, courses superiorly, and then runs adjacent to CN III and IV as they run through the dura and provide them with blood supply. In this specimen, the tentorial artery is originating from the ILT, which courses lateral to the tentorium to contribute to the blood supply of the interdural segment of the trochlear nerve. **(B)** The dura of the lateral wall of the left CS has been removed to show the intracavernous CNs and terminal branches of the ILT. The ILT giving off the superior orbital branch, which goes through the superior orbital fissure and supplies blood to the distal segments of all CNs passing through the superior orbital fissure. The ILT then courses laterally over V1 and gives off the artery of the foramen rotundum, which supplies the distal segment of V2. **(C)** Microscopic view of the lateral wall of left cavernous sinus showing terminal branches of the ILT. The ILT giving off the superior orbital branch, and the superior branch. It then courses laterally under V2 segment and gives off the artery of foramen rotundum. The ILT also gives rise to tentorial branch which is supplying CN IV. CN, cranial nerve; CS, cavernous sinus; ICA, internal carotid artery; ILT, inferolateral trunk; MHT, meningohypophyseal trunk; SLT, superolateral trunk.

The MHT, the largest and most constant branch of the cavernous ICA, arises from the short vertical segment of the intracavernous ICA, close to the posterior genu, in 20% of the specimens studied. In the other 80% of specimens, the MHT arises from the middle third of the posterior genu. It typically gives rise to three branches: 1) the inferior hypophyseal, which travels medially to supply the posterior lobe of the pituitary gland; 2) the tentorial artery (known as the artery of Bernasconi-Cassinari), which courses lateraly towards the tentorium to contribute to the blood supply of the interdural segment of the oculomotor and trochlear nerves; and 3) the dorsal meningeal artery (also called the lateral clival artery), which passes posteriorly through the CS to supply the clival dura and abducens nerve within Dorello’s canal. This artery usually divides into medial and lateral branches. The medial branch accompanies the abducens nerve into Dorello’s canal and sometimes anastomoses with the clival ramus of the jugular branch of the ascending pharyngeal artery (**Figures 1A–C**).

**Figure 2:**
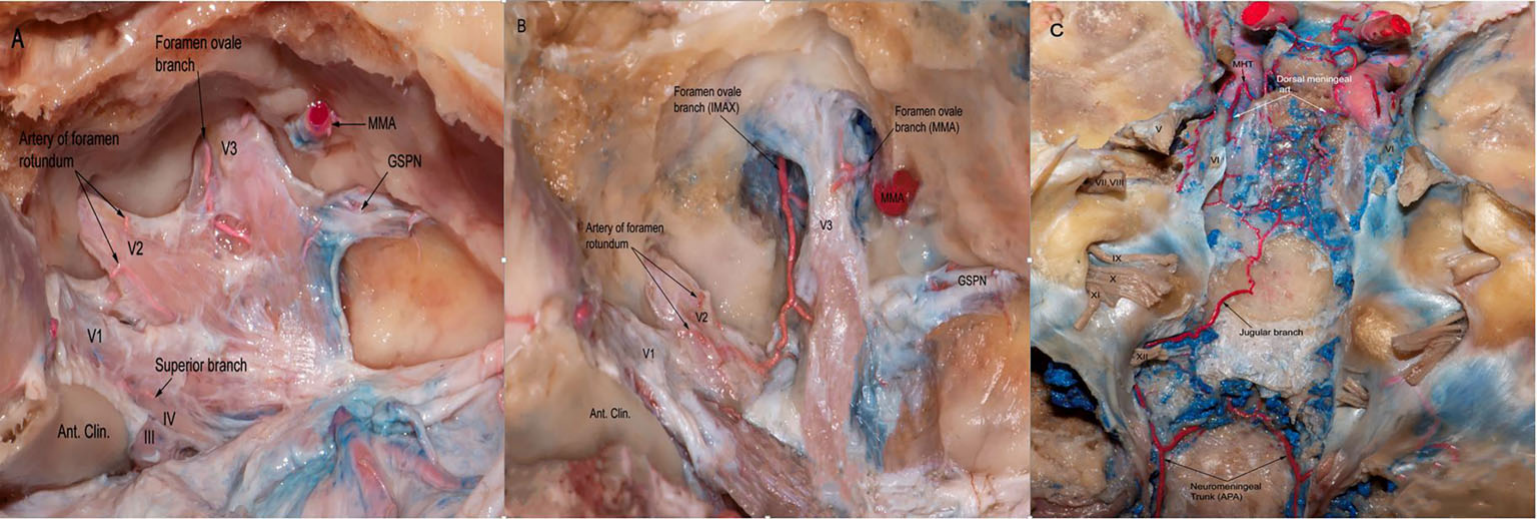
**(A)** The dura of the lateral wall of the right CS has been removed to show the terminal branches of the ILT providing blood supply to CN III,IV as well asV1,V2,V3 branches of the trigeminal nerve. ILT giving off the superior branch, the artery of foramen rotundum, and the foramen ovale branch. **(B)** Another view from the same specimen after careful drilling of the foramen ovale showing the anastomotic network between the terminal branches of ILT and branches from the internal maxillary artery. Note the branches arising from MMA supplying the V3 segment of the trigeminal nerve. **(C)** Posterior view of the clivus in a colored silicone-injected human cadaveric specimen showing a rich anastomotic network between the dorsal meningeal branches arising from MHT and the jugular branches arising from neuromeningeal trunk, which is a branch of the ascending pharyngeal artery. APA, ascending pharyngeal artery; MMA, medial meningeal artery.

The ILT is present in 85% of the specimens. It arises from the lateral side of the midportion of the horizontal segment of the intracavernous ICA, and courses inferiorly. The ILT arises directly from the carotid artery in 80% of the specimens and from the MHT in 5%. The ILT typically divides into three to four branches: 1) the superior orbital branch (also called the antero-medial branch), which runs to the superior orbital fissure; 2) the foramen rotundum artery (known as the antero-Iateral branch), typically accompanies the maxillary nerve (V2) through the foramen rotundum, supplies the V2 segment of the trigeminal nerve, and anastomoses with the internal maxillary artery (IMAX); 3) the foramen ovale branch, which travels with the mandibular (V3) segment of the trigeminal nerve through the foramen ovale and has anastomoses with the IMAX and/or petrosal branch of the middle meningeal artery; 4) the superior branch of the ILT, which follows the III and IV nerve along the lateral wall of the CS (**Figures 2A–C**).

In 10% of the specimens a superolateral trunk is found to arise from the supero-lateral aspect of the horizontal segment of the intracavernous ICA, it directs superiorly, and then runs adjacent to the oculomotor and trochlear cranial nerves and provides them with blood supply at their dural entrance. McConnell’s capsular artery is present in 20% of the cases and arises from the medial aspect of the horizontal segment of the intracavernous ICA and courses toward the capsule of the pituitary gland, where it anastomoses with the opposite artery. The capsular artery does not provide any blood supply to the CNs.

**Figure 3:**
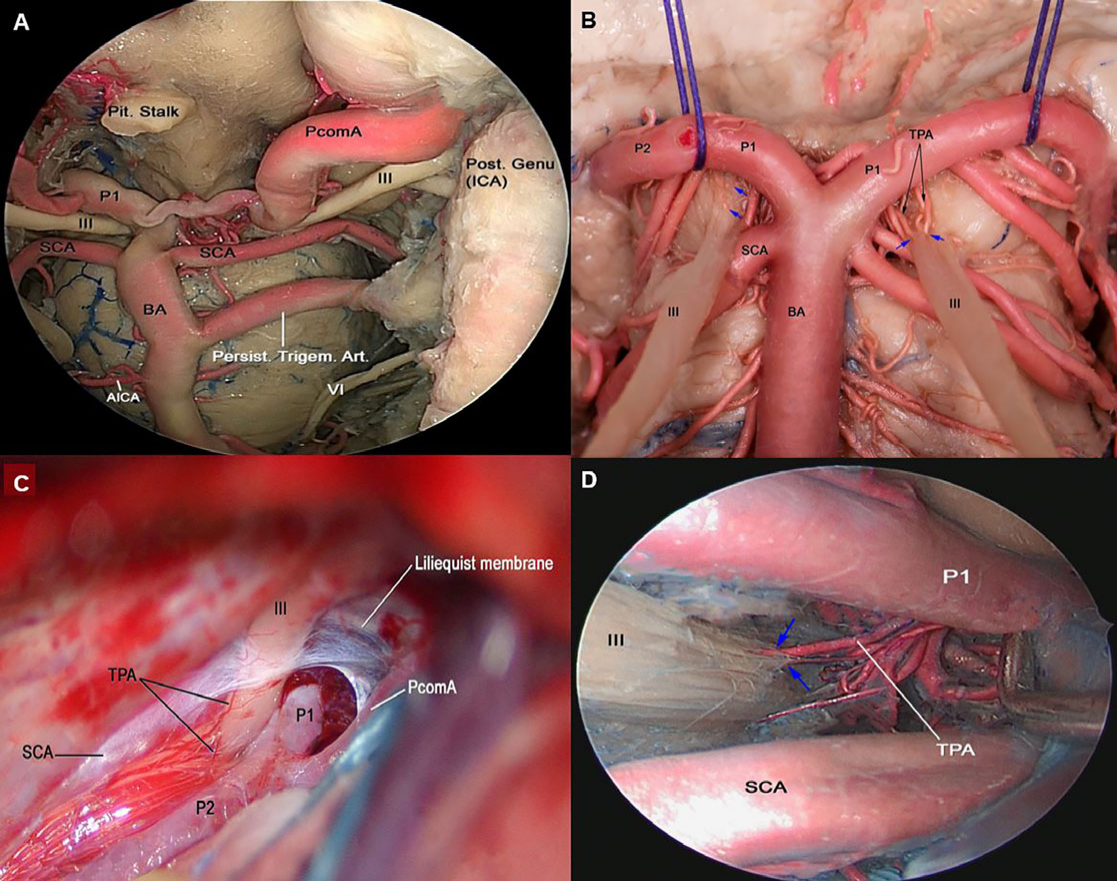
**(A)** Endoscopic view in a colored silicone-injected human cadaveric specimen showing a persistent trigeminal artery anastomosing with the left intracavernous ICA with the basilar artery between SCA and AICA origins. **(B)** Cross-section of midbrain at the level of the superior colliculus demonstrating the course of cisternal segment of the III nerve, which passes between PCA and SCA. Note the thalamoperforating branches arising from P1 supplying the cisternal segment (blue arrow). BA, basilar artery; PCA, posterior cerebral artery; SCA, superior cerebellar artery **(C)** Left trans-sylvian exposure of the ambient cistern, revealing: the PCA and SCA perforating branches supplying the cisternal segment of the III nerve. **(D)** Endoscopic view of the interpeduncular region, showing the cisternal segment of right CN III. TPA arising from pre-communicating segment of PCA (P1) supplying the cisternal segment of CN III are shown (blue arrow). AICA, anterior inferior cerebellar artery; CN, Cranial Nerve; PCA, posterior cerebral artery; PcomA, posterior communicating artery; SCA, Superior Cerebellar Artery; TPA, Thalamoperforating Arteries.

In one specimen (5%) a persistent trigeminal artery is identified. It arises from the middle third of the posterior genu of the intracavernous ICA, courses backward through the posterior wall of the CS lateral to Dorello’s canal and medial to Meckel’s cave to join the basilar artery between the origin of the superior cerebellar artery and the anterior inferior cerebellar artery (**Figure 3A**). In this particular case, the MHT and its three branches originate from the persistent trigeminal artery.

### Blood Supply of the Intracavernous Nerves

#### The Oculomotor Nerve

The oculomotor nerve is supplied by the ILT branches in all studied specimens. The proximal cavernous segment of the oculomotor CN receives its blood supply through the superior branch of the ILT in 85% of the specimens and the tentorial artery of the MHT in 15%. In addition, the proximal segment of the III CN receives blood supply from the superolateral trunk in 10% of the cases; the distal segment of the III CN is supplied exclusively by the superior orbital branch of the ILT (**Figure 1** and **Table 1**). The superior orbital branch of the ILT supplies the distal segments of all CNs passing through the superior orbital fissure. The cisternal segment of the oculomotor nerve receives blood supply from the thalamoperforating arteries that originate from the P1 and the proximal P2 segments of the posterior cerebral artery (**Figures 3B–D**).

**Table 1:** The Blood Supply of Intracavernous Cranial Nerves: Microscopic and Endoscopic perspective.

Cranial nerve		Blood supply	Frequency	Transcranial perspective	Endoscopic endonasal perspective
**Oculomotor nerve**	Proximal segment	Proximal superior branch (ILT)	85%	Lateral wall of the CS	No visible
		Tentorial artery (MHT)	15%	No visible	Posterior compartment of the CS
		Superior lateral trunk	10%	Lateral wall of the CS	No visible
	Distal segment	Superior orbital branch (ILT)	85%	Lateral wall of the CS	No visible
**Trochlear nerve**	Proximal segment	Proximal superior branch (ILT)	75%	Lateral wall of the CS	No visible
		Tentorial artery (MHT)	25%	Lateral wall of the CS	Superior compartment of the CS
	Distal segment	Superior orbital branch (ILT)	85%	Lateral wall of the CS	No visible
**Abducens nerve**	Proximal third	Dorsal meningeal artery (MHT)	100%	Posterior wall of the CS	Posterior compartment of the CS
	Middle third	ILT branches	85%	Lateral wall of the CS	Inferior and lateral compartment of the CS
	Distal third	Superior orbital branch (ILT)	85%	Lateral wall of the CS	No visible
**Ophthalmic branch**	Proximal segment	ILT branches	85%	Lateral wall of the CS	No visible
	Distal segment	Superior orbital branch. (ILT)	85%	Lateral wall of the CS	
**Maxillary branch**	Proximal segment	ILT branches	85%	Lateral wall of the CS	Lateral compartment of the CS
	Distal segment	Foramen rotundum artery (ILT)	80%	Lateral wall of the CS	
**Gasserian ganglion**	Medial half	ILT and/or Tentorial artery (MHT)	100%		
	Lateral half	ILT and/or Dorsal branches (MMA)	100%		

CS, cavernous sinus; MHT, meningohypophyseal trunk; MMA, medial meningeal artery; ILT, inferolateral trunk.

#### The Trochlear Nerve

The proximal trochlear nerve (IV) receives its vascularity from the superior branch of the ILT and from the tentorial artery in 75% and 25% of the specimens, respectively (**Figures 1A–C**); when the superior branch is present, it usually curves superiorly and posteriorly and follows the trochlear nerve along the lateral wall of the CS. The distal segment of the trochlear nerve is exclusively supplied by the superior orbital branch of the ILT.

#### The Abducens Nerve

In all of the specimens studied the proximal third of the abducens nerve (VI), at the level of Dorello’s canal, receives blood supply exclusively from the dorsal meningeal artery arising from the MHT. The middle and distal thirds of the abducens nerve are vascularized by branches of the ILT. When the ILT is present, it crosses over the middle third segment of the abducens nerve. This constant relationship between the ILT and the 6^th^ CN is a reliable surgical landmark for the identification of the abducens nerve when performing surgery in this region.

#### The Trigeminal Nerve

The proximal ophthalmic (V1) and proximal maxillary (V2) segments of the trigeminal nerve are also vascularized by small branches of the ILT or its equivalent in 100% of the specimens. The distal ophthalmic segment of the trigeminal nerve is also supplied by the superior orbital branch of the ILT, while the distal maxillary segment is supplied by the artery of the foramen rotundum, arising from the ILT.

The medial side of the Gasserian ganglion receives its vascularity from the small branches of the ILT and the tentorial artery; the lateral side is supplied by branches from the artery of the foramen rotundum and the dorsal branches of the middle meningeal artery.

**Figure 4:**
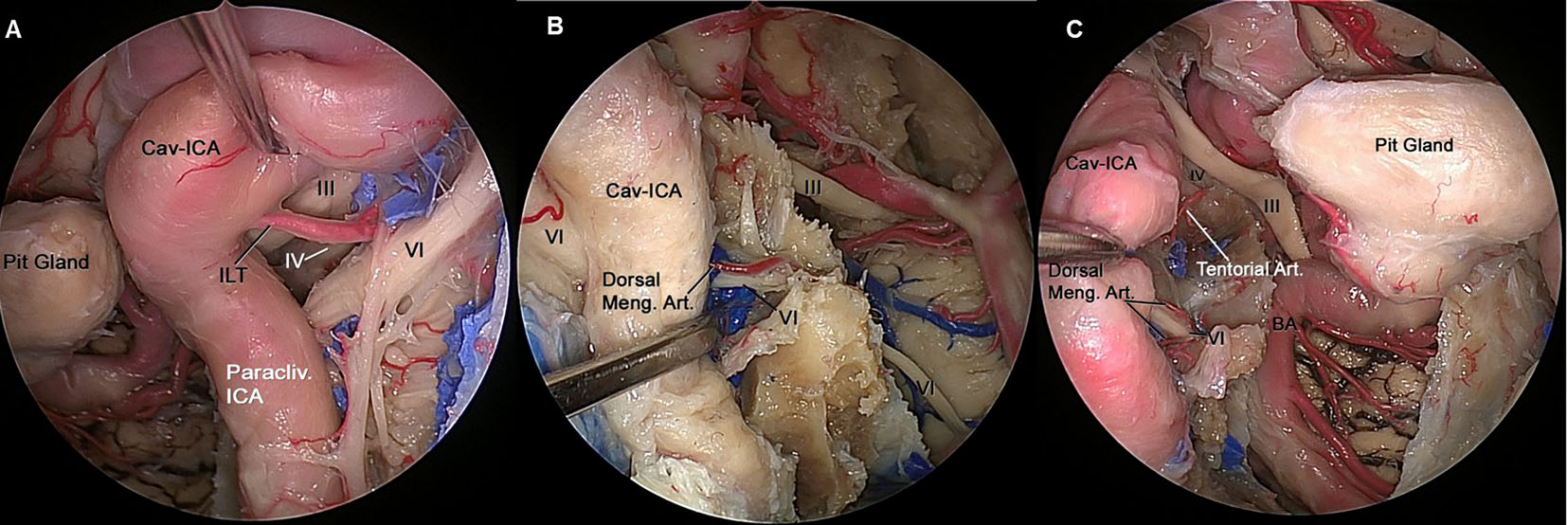
**(A)** Endoscopic view of left CS showing the neurovascular relationships in the lateral compartment. The ILT arising from the midportion of the horizontal segment of the intracavernous ICA, and its branches can be identified running from medial to lateral where they distribute along the lateral wall of the CS and supplies to the of CN III, CN IV and distal CN VI. **(B)** The paraclival ICA is retracted laterally to expose CN VI inside Dorelo’s canal. Dorsal meningeal artery (Dorsal Meng. Art.), a branch from MHT, which is located in the posterior compartment of cavernous sinus and supply proximal segment of CN VI at the level of Dorelo’s canal. **(C)** Endoscopic view of right CS showing the neurovascular relationships in the superior and posterior compartment of the CS. The Cav-ICA is retracted laterally. The tentorial artery and dorsal meningeal artery can be identified running in the superior and posterior compartment of the CS, respectively. The dorsal meningeal artery supplying proximal segment of CN VI at the level of Dorelo’s canal and the tentorial artery supplying the CN IV are shown. BA, Basilar Artery; Cav, Cavernous; CAV-ICA, Cavernous Internal Carotid Artery; CN, Cranial Nerve; Dorsal Meng. Art, Dorsal Meningeal Artery; ICA, Internal Carotid Artery; ILT, inferolateral trunk.

All ILT branches end on the inferomedial aspects of the intracavernous CNs. The MHT branches are located in the posterior compartment of the CS. From a transcranial perspective, all intracavernous ICA branches supplying the intracavernous CNs are found in the lateral wall of the CS, except the dorsal meningeal artery, which is located in the posterior wall. From an endoscopic endonasal perspective, the tentorial artery and dorsal meningeal artery are found in the superior compartment and posterior compartment of the CS, respectively. The ILT is found in the lateral and inferior compartments of the CS (**Figures 4A–C**).

### Case Illustrations

A tumor with CS invasion is selected to illustrate the surgical application of these findings.

#### CS Meningioma

**Figure 5:**
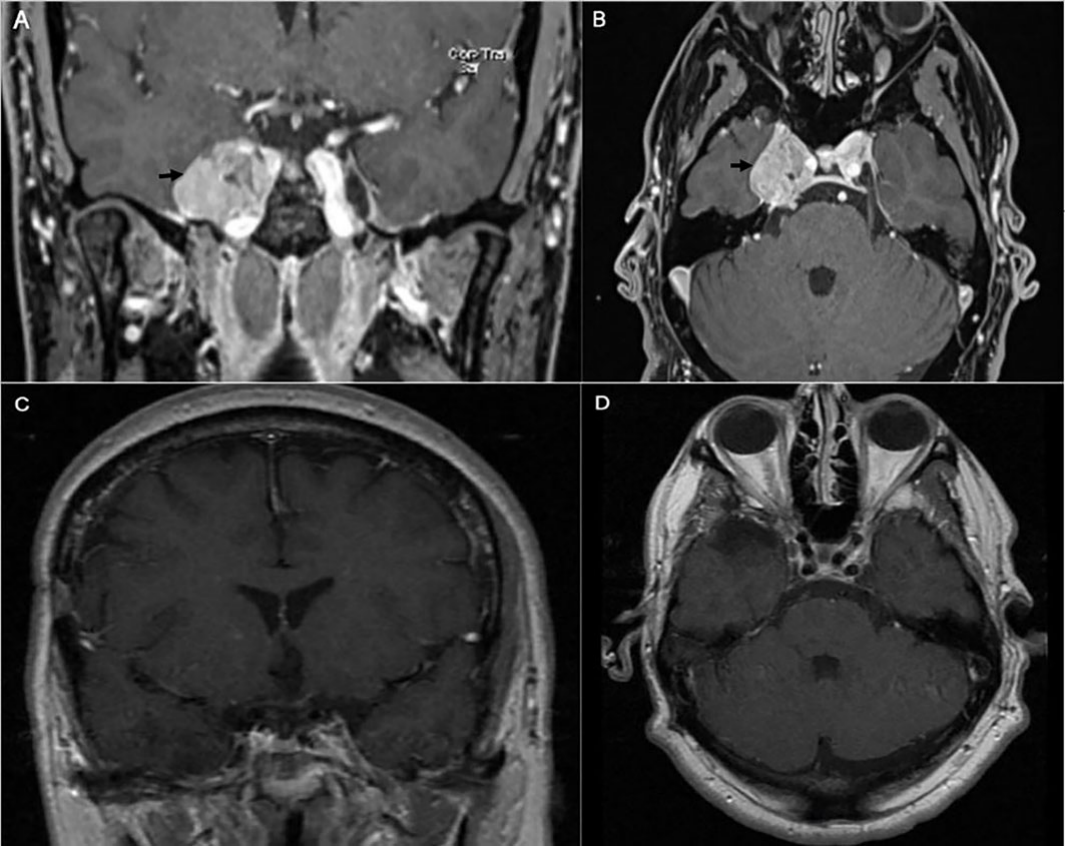
Preoperative coronal **(A)** and axial **(B)** brain MRI with contrast showing heterogeneous enhancing tumor involving the right cavernous sinus (arrow). The patient is known to have right cavernous sinus meningioma for which he had stereotactic radiosurgery 4 years prior to presentation. Postoperative coronal **(C)** and axial **(D)** brain MRI with contrast showing gross total excision of the meningioma.

A 43-year-old right-handed male had an initial presentation of right facial numbness. An MRI scan of his brain showed a right CS tumor consistent with a meningioma. The patient underwent stereotactic radiosurgery at an outside hospital. His facial numbness gradually improved. Four years later, he had recurrence of the right facial numbness in the V3 distribution with associated right eyelid ptosis and diplopia. On neurological examination he had decreased sensation to light touch in the three branches of the trigeminal nerve. He had a right eyelid ptosis and a six-nerve palsy. A new MRI scan of the brain showed radiological progression of the tumor (**Figures 5A, B**). An orbitozygomatic craniotomy was performed and gross total tumor resection was achieved. The facial numbness he had preoperatively gradually improved, and his extraocular movements and eyelid ptosis recovered completely. An MRI revealed a gross total resection of the tumor. The histopathology showed a clear cell grade 2 meningioma. Postoperatively He received fractionated radiation therapy to the surgical bed. He remained tumor free at his last follow-up four years after his surgery (**Figures 5C, D**).

## Discussion

Lesions involving the CS and lateral sellar region, including tumors, vascular lesions, infections, and inflammatory pathologies have an intimate relationship with the intracavernous CNs and their blood supply originating from the intracavernous ICA. Despite a decrease in the morbidity and mortality rates associated with CS surgery, CN deficits remain the most frequent cause of disability following CS surgery (1–4, 9). In this study we investigate the anatomic variations of the intracavernous CNs blood supply when exposed from a microsurgical transcranial as well as endoscopic endonasal perspectives. This relevant information can be applied or endoscopic and microsurgical approaches of the cavernous sinus.

The underlying mechanisms of intraoperative nerve injury is thought to be multimodal. Although ischemic insults secondary to devascularization of the cavernous segment of the cranial nerves might cause cranial nerve deficits, usually those deficits are transient as the ensuing ischemic insult mainly affects the myelin sheet of the nerve which tends to regenerate within weeks. This is a process similar to what is seen in peripheral nerves that can be extensively dissected and mobilized with minimal or transient nerve dysfunction. In contrast a more proximal, cisternal ischemic injury to the same cranial nerves might also affect their nuclei causing a more permanent deficit. Direct injury to the intracavernous cranial nerves is usually a cause of permanent deficit. Such injuries can be from a direct mechanical trauma or from thermal insults secondary to bipolar coagulation (10, 12, 15). This is where a thorough understanding of the cranial nerves blood supply becomes important as precise control of those vessels during cavernous sinus surgery will preserve a bloodless field and avoid cranial nerve injuries. In order to avoid permanent injury to the CNs, a dissection of the CNs has to be tangential to the nerves along a longitudinal axis. When the CNs are released in this way, they can tolerate a certain amount of manipulation without permanent deficits.

Our findings are in concordance with the study conducted by Krisht et al. (1) and Harris et al. (6) We describe the details of the most common anatomic patterns of blood supply to the intracavernous CNs and observed that the ILT branches play an important role in vascularizing the III, IV, V, and VI CNs as they pass into the CS and the superior orbital fissure, and through the foramen rotundum. Furthermore, we observed that the dorsal meningeal artery branch of the MHT provides blood supply to the proximal third of the abducens nerve at the level of Dorello’s canal. The ILT also provides vascularity to the mesial surface of the Gasserian ganglion as it anastomoses with the artery of the foramen ovale, originating from the internal maxillary artery, which in turn provides blood supply to V3 and the lateral aspect of the Gasserian ganglion.

The ILT typically arises from the lateral aspect of the intracavernous ICA and usually supplies the intracavernous CNs, with extensive anastomoses with the extracranial circulation, particularly branches of the IMAX, the middle meningeal artery, and the ophthalmic artery (**Figures 2A, B**). These anastomoses, mainly the superior orbital fissure branch anastomoses with the deep recurrent meningeal branch of the ophthalmic artery, are an important consideration during embolization (16–18).

We also observed that the dorsal meningeal artery (medial branch) arising from the MHT anastomoses with the clival ramus of the jugular branch of the ascending pharyngeal artery (**Figure 2C**)

All ILT trunk branches were located on the inferomedial aspects of the intracavernous CNs; the MHT branches were located on the posterior compartment of the CS. From a transcranial perspective, all intracavernous ICA branches that supplied blood to the intracavernous CNs were found in the lateral wall of the CS, except the dorsal meningeal artery, which was located in the posterior wall. From an endoscopic endonasal perspective, the tentorial artery and the dorsal meningeal artery were found in the superior and posterior compartments of the CS, respectively. The ILT was found in the lateral and inferior compartments of the CS.

Of surgical interest, we observed that each foramen had an artery, which are summarized as follows: the superior orbital branch, which passed through the superior orbital fissure and supplied blood to the distal segments of all of the CNs passing through to it; the artery to the foramen rotundum, another very important vessel, which supplies the V2 segment of the trigeminal nerve; the foramen ovale branch supplies the V3 segment of the trigeminal nerve (**Figure 2A**); and the dorsal meningeal artery, which ran into Dorello’s canal and provided blood supply to the abducens nerve within Dorello’s canal (**Figure 1**).

Beyond studying the blood supply of CNs in the CS from both transcranial and endoscopic endonasal perspectives the study presented here describes the blood supply to the cisternal III nerve and the anastomoses of the intracavernous ICA’s branches with the external carotid artery. In addition, we report for the first time that the superolateral trunk can provide blood supply to the III and IV CNs, as found in 10% of our specimens (**Figures 1A, B**).

Based on the above findings we were able to identify 3 vascular zones to the cranial nerves passing through the cavernous sinus: Zone 1 being the cisternal segment of the cranial nerves, receiving blood supply from the vertebrobasilar system. Zone 2 being the intracavernous segment of the cranial nerves receiving their blood supply from the internal carotid artery and Zone 3, the extracranial portion of those cranial nerves and receiving blood supply from branches of the external carotid artery. As such Zone 1 of those cranial nerves is most sensitive to ischemic insults and Zones 2 and 3 are more resilient. For these reasons experts recommend avoiding the manipulation of the proximal cisternal segment of the III nerve as it arises from the midbrain of the brainstem as manipulation at this level can cause a permanent deficit.

Certainly, several other anatomic variations may exist in the intracavernous ICA’s branch anatomy and the CNs’ blood supply, even among patients without CS disease. In addition, tumors involving the CS, once a certain size, may cause significant distortion of the region’s microanatomy, posing an additional surgical challenge. Therefore, a detailed understanding of the blood supply of the intracavernous CNs is essential for improving outcomes when operating on lesions involving the CS.

**Figure 6:**
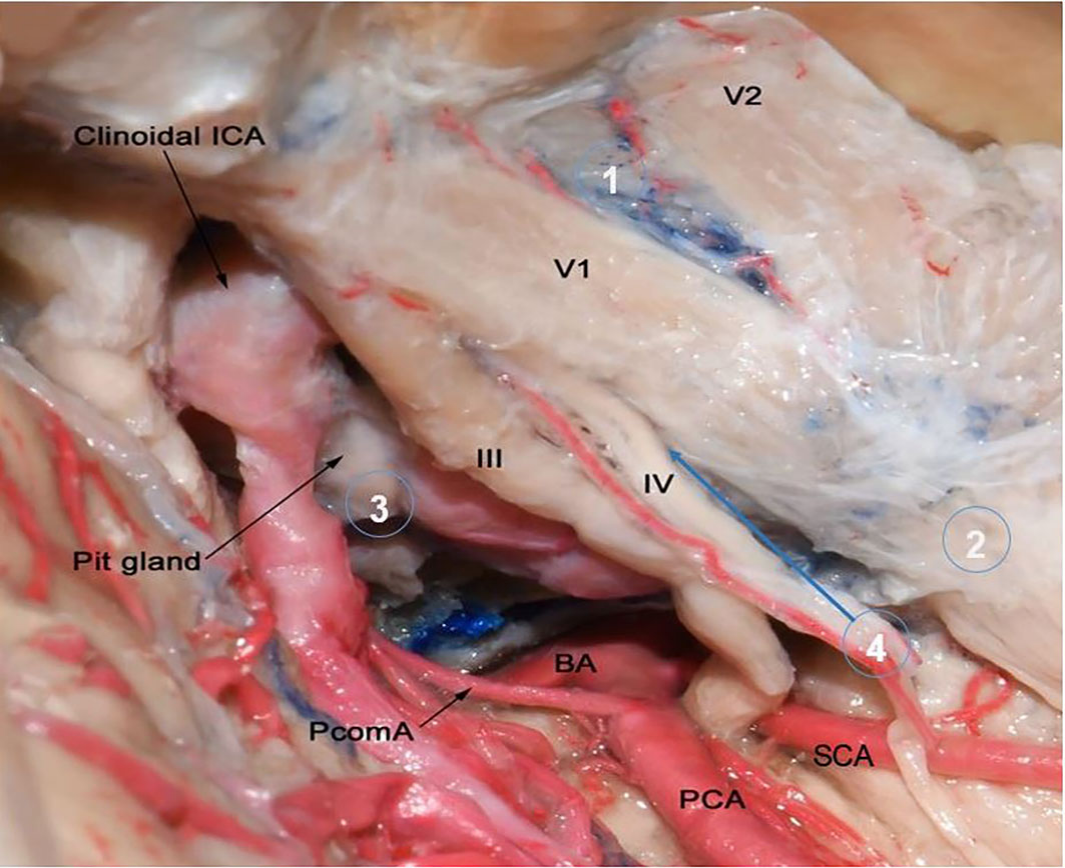
Microscopic lateral view of right cavernous sinus to show the origin of most common tumors that involve the cavernous sinus. Circle number 1 is over the lateral wall of cavernous sinus which is the most common origin of CS meningiomas and they get their blood supply from MHT and ILT (not shown in this image). They account around 41% of all cavernous sinus tumors. Circle 2 is over trigeminal nerve which is the origin of schwannomas. Trigeminal schwannomas are the second most common cavernous sinus tumors and 49% originate in middle cranial fossa (19). They receive their blood supply from MHT and branches from external carotid artery. Circle 3 is over pituitary gland. Pituitary adenomas are the most common tumors that may extend into the cavernous sinus with an incidence of 10%. Their blood supply obtained from superior and inferior hypophyseal arteries. Circle 4 points to intracavernous sinus compartment. Cavernous hemangiomas are benign vascular tumors which are the most common primary intracavernous lesion. Metastasis is also may be seen in intracavernous compartment. Tumors originating from intracavernous compartment may get their blood supply from all intracavernous ICA branches. BA, Basilar Artery; ICA, Internal Cerebral Artery; PCA, Posterior Cerebral Artery; PcomA, Posterior Communicating Artery; Pit, Pituitary; SCA, Superior Cerebellar Artery.

The most common tumor affecting the cavernous sinus is meningioma and accounts for 41% of cavernous sinus tumors (**Figure 6**) (20). It originates from arachnoid cap cells of CNs in the lateral wall of cavernous sinus (21). Blood supply of CS meningiomas are from external carotid artery branches (the same of middle cranial fossa dura blood supply) and branches from MHT and ILT (22, 23). However, meningiomas originating from nearby regions (e.g. petroclival region, medial sphenoid wing) may extend into CS and may have a different blood supply. Other common tumor affecting cavernous sinus is trigeminal schwannomas. They are the second most common intracranial schwannomas after vestibular schwannoma with an incidence of 1-8% of all intracranial schwannomas (19). Trigeminal nerve schwannomas receive their blood supply through MHT or branches from external carotid artery (24). Also, pituitary adenomas are common to invade the CS by extending through its medial wall and 6%-10% of all pituitary adenomas invade the CS (25). They supplied by the medial branches of ICA, namely the superior and inferior hypophyseal trunks.

## Conclusion

ILT branches provide blood supply to all intracavernous CNs except the proximal segment of the abducens nerve at the level of Dorello’s canal. MHT branches supply the proximal segment of the abducens nerve and the medial aspect of the Gasserian ganglion. Middle meningeal artery branches supply the lateral aspect of the Gasserian ganglion. This study demonstrated extensive anastomoses between ILT branches and branches arising from the external carotid artery. Understanding the anatomy of the intracavernous ICA’s branches is important to improving surgical outcomes with tumors involving the CS.

## Data Availability Statement

The raw data supporting the conclusions of this article will be made available by the authors, without undue reservation.

## Ethics Statement

This study was reviewed and approved by the Ethical Committee at Cleveland Clinic Florida.

## Author Contributions

EN: data collection, data analysis, writing the manuscript. BI: data collection, data analysis, writing the manuscript. BM: data collection, writing and review the manuscript. AA: writing and review the manuscript. CS: revising the manuscript. MO: contributed to study design, critically revised the manuscript. HB-R: contributed to study design, critically revised the manuscript. BA: study design, supervised the whole study and critically revised the manuscript. All authors contributed to the article and approved the submitted version.

## Supplementary Material

The Supplementary Material for this article can be found online at: https://www.frontiersin.org/articles/10.3389/fonc.2021.702574/full#supplementary-material

## Conflict of Interest

The authors declare that the research was conducted in the absence of any commercial or financial relationships that could be construed as a potential conflict of interest.

## Publisher’s Note

All claims expressed in this article are solely those of the authors and do not necessarily represent those of their affiliated organizations, or those of the publisher, the editors and the reviewers. Any product that may be evaluated in this article, or claim that may be made by its manufacturer, is not guaranteed or endorsed by the publisher.

## References

[1] Al-Mefty O Smith RR. Surgery of Tumors Invading the Cavernous Sinus. Surg Neurol (1988) 30(5):370–81. doi: 10.1016/0090-3019(88)90200-5 3187882

[2] Dolenc V. Direct Microsurgical Repair of Intracavernous Vascular Lesions. J Neurosurg (1983) 58(6):824–31. doi: 10.3171/jns.1983.58.6.0824 6854374

[3] Krisht A Barnett DW Barrow DL Bonner G. The Blood Supply of the Intracavernous Cranial Nerves: An Anatomic Study. Neurosurgery (1994) 34(2):275–9. doi: 10.1227/00006123-199402000-00011 8177389

[4] Fernandez-Miranda JC Zwagerman NT Abhinav K Lieber S Wangm EW Snyderman CH. Cavernous Sinus Compartments From the Endoscopic Endonasal Approach: Anatomical Considerations and Surgical Relevance to Adenoma Surgery. J Neurosurg (2017) 129(2):430–41. doi: 10.3171/2017.2.JNS162214 28862552

[5] Dolenc VV Kregar T Ferluga M Fettich M Mozrina A. Treatment of Tumors Invading the Cavernous Sinus. In: Dolenc VV, editor. The Cavernous Sinus. Wien New York: Springer (1987). p. 377–91.

[6] Lasjaunias P Moret J Manelfe C Theron J Hasso T Seeger J. Arterial Anomalies at the Base of the Skull. Neuroradiology (1977) 13(5):267–72. doi: 10.1007/BF00347072 896036

[7] Parkinson D Shields CB. Persistent Trigeminal Artery: Its Relationship to the Normal Branches of the Cavernous Carotid. J Neurosurg (1974) 40(2):244–8. doi: 10.3171/jns.1974.40.2.0244 4809123

[8] Harris FS Rhoton AL. Anatomy of the Cavernous Sinus. A Microsurgical Study. J Neurosurg (1976) 45(2):169–80. doi: 10.3171/jns.1976.45.2.0169 939976

[9] Knosp E Müller G Perneczky A. The Blood Supply of the Cranial Nerves in the Lateral Wall of the Cavernous Sinus. In: The Cavernous Sinus. Vienna: Springer (1987). p. 67–80.

[10] Tekdemir I Tüccar E Çubuk HE Ersoy M Elhan A Deda H. Branches of the Intracavernous Internal Carotid Artery and the Blood Supply of the Intracavernous Cranial Nerves. Ann Anat (1998) 180(4):343–8. doi: 10.1016/S0940-9602(98)80040-X 9728276

[11] Tran-Dinh H. Cavernous Branches of the Internal Carotid Artery: Anatomy and Nomenclature. Neurosurgery (1987) 20(2):205–10. doi: 10.1227/00006123-198702000-00001 3561725

[12] Willinsky R Lasjaunias P. Intracavernous Branches of the Internal Carotid Artery. Surg Radiol Anat (2005) 9(3):201–15. doi: 10.1007/BF02109631 3122341

[13] d’Avella E Tschabitscher M Santoro A Delfini R. Blood Supply to the Intracavernous Cranial Nerves: Comparison of the Endoscopic and Microsurgical Perspectives. Oper Neurosurg (2008) 62(suppl_5):ONS305–11. doi: 10.1227/01.neu.0000326011.53821.ea 18596508

[14] Alfieri A Jho HD. Endoscopic Endonasal Cavernous Sinus Surgery: An Anatomic Study. Neurosurgery (2001) 48(4):827–36; discussion 836–7. 11322443

[15] Aydin S Cavallo LM Messina A Dal Fabbro M Cappabianca P Barlas O. The Endoscopic Endonasal Trans-Sphenoidal Approach to the Sellar and Suprasellar Area. Anatomic Study. J Neurosurg Sci (2007) 51:129–38. 17641577

[16] Conti M Prevedello DM Madhok R Faure A Ricci UM Schwarz A. The Antero-Medial Triangle: The Risk for Cranial Nerves Ischemia at the Cavernous Sinus Lateral Wall: Anatomic Cadaveric Study. Clin Neurol Neurosurg (2008) 110(7):682–6. doi: 10.1016/j.clineuro.2008.04.007 18554776

[17] Toma N. Anatomy of the Ophthalmic Artery: Embryological Consideration. Neurol Med Chirurg (2016) 56(10):585–91. doi: 10.2176/nmc.ra.2015-0324 PMC5066078 27298261

[18] Bertelli E Regoli M Bracco S. An Update on the Variations of the Orbital Blood Supply and Hemodynamic. Surg Radiol Anat (2017) 39(5):485–96. doi: 10.1007/s00276-016-1776-9 PMC5406424 27830321

[19] Samii M Migliori MM Tatagiba M Babu R. Surgical Treatment of Trigeminal Schwannomas. J Neurosurg (1995) 82(5):711–8. doi: 10.3171/jns.1995.82.5.0711 7714594

[20] Amelot A van Effenterre R Kalamarides M Cornu P Boch AL. Natural History of Cavernous Sinus Meningiomas. J Neurosurg (2018) 1:1–8. doi: 10.3171/2017.7.JNS17662 29600913

[21] Kehrli P Maillot C Wolff Quenot MJ. Sheaths of Cranial Nerves in the Lateral Wall of the Cavernous Sinus. An Embryological and Anatomical Study. Neurochirurgie (1995) 41:403−12. 8815415

[22] Robinson DH Song JK Eskridge JM. Embolization of Meningohypophyseal and Inferolateral Branches of the Cavernous Internal Carotid Artery. AJNR Am J Neuroradiol (1999) 20(6):1061–7. PMC7056238 10445445

[23] Barr JD Mathis JM Horton JA. Iatrogenic Carotid-Cavernous Fistula Occurring After Embolization of a Cavernous Sinus Meningioma. AJNR Am J Neuroradiol (1995) 16(3):483–5. PMC8337657 7793369

[24] Abramowitz J Dion JE Jensen ME Lones M Duckwiler GR Viñuela F. Angiographic Diagnosis and Management of Head and Neck Schwannomas. AJNR Am J Neuroradiol (1991) 12(5):977–84. PMC8333487 1950934

[25] Cottier JP Destrieux C Brunereau L Bertrand P Moreau L Jan M. Cavernous Sinus Invasion by Pituitary Adenoma: MR Imaging. Radiology (2000) 215(2):463–9. doi: 10.1148/radiology.215.2.r00ap18463 10796926

